# Mapping Cell Phenomics with Multiparametric Flow Cytometry Assays

**DOI:** 10.1007/s43657-021-00031-0

**Published:** 2022-01-09

**Authors:** Yang Liu, Haichu Zhao, Boqiang Fu, Shan Jiang, Jing Wang, Ying Wan

**Affiliations:** 1 Biomedical Analysis Center, Army Medical University, Chongqing, 400038 China; 2 Chongqing Key Laboratory of Cytomics, Chongqing, 400038 China; 3 Institute for Advanced Study, Shenzhen University, Shenzhen, 518055 China; 4 National Institute of Metrology, Beijing, 100029 China

**Keywords:** Cell phenomics, Multiparametric cytometry, Flow cytometry assay

## Abstract

Phenomics explores the complex interactions among genes, epigenetics, symbiotic microorganisms, diet, and environmental exposure based on the physical, chemical, and biological characteristics of individuals and groups. Increasingly efficient and comprehensive phenotyping techniques have been integrated into modern phenomics-related research. Multicolor flow cytometry technology provides more measurement parameters than conventional flow cytometry. Based on detailed descriptions of cell phenotypes, rare cell populations and cell subsets can be distinguished, new cell phenotypes can be discovered, and cell apoptosis characteristics can be detected, which will expand the potential of cell phenomics research. Based on the enhancements in multicolor flow cytometry hardware, software, reagents, and method design, the present review summarizes the recent advances and applications of multicolor flow cytometry in cell phenomics, illuminating the potential of applying phenomics in future studies.

## Introduction

Phenomics is the systematic study of all phenotypes of an organism or cell under environmental conditions at the genomic level (Furbank and Tester 2011). Phenomics is a powerful approach that can depict the aggregate phenotype of organisms under the influence of genetic and environmental factors (Varki and Altheide 2005). Single-cell phenomics data fully reflect the characteristics of cells, including subcellular structures from digital images, temporal attributes of cellular features, and statistical analysis of high-dimensional phenotyping (Ohya et al. 2015). Cell phenomics uniquely allows researchers to determine how genomic variants affect phenotypes, thus providing deep insights into the causes of disease.

Flow cytometry can measure multiple individual features per cell for tens of thousands of cells simultaneously, providing a quantitative and qualitative assessment of cells and molecular states in tissue, organs, or other biosystems (McKinnon 2018). By means of fluorescence staining, flow cytometry can include qualitative and quantitative analysis at the single-cell level for DNA, antigens and receptors, ion concentrations, and enzyme activities. Flow cytometry rapidly selects target cells with common phenotypic characteristics from a large cell population, and it has become a critical cytomic technique for describing cell characteristics.

With the development of multicolor flow cytometry, there has been a rapid expansion in measurement capacity, with up to 60 parameters now routinely measured in a single cell. Flow cytometry can therefore broaden the dimensions of cell phenotyping and provide new solutions and possibilities in the field of cell phenomics. In the present review, we summarize the technological progress of flow cytometry in the past 50 years, including advances in optical and electronic technology, fluorophore synthesis technology, data analysis methods, and automated equipment standardization, and discuss the current and prospective application of multicolor flow cytometry in cell phenotyping and cell phenomics.

## Developments in Hardware, Software, and Reagents for Cell Phenomics

### Advances in Optical and Electronic Technology to Improve the Resolution of Cell Phenomics

#### Multicolor Conventional Flow Cytometry

The laser configuration of a flow cytometer determines the set of applicable fluorescence signals. When laser-equipped flow cytometers were first developed, argon-ion and helium–neon gas lasers were used to provide 488 and 633 nm laser lines, respectively (Oi et al. 1982; Shapiro and Stephens 1986; Shapiro 1993). Since the 1990s, laser diode (LD) and diode-pumped solid-state (DPSS) lasers have been rapidly developed and improved, as solid-state lasers exhibit significant advantages over gas lasers in terms of wavelength availability, component dimensions, and life span and thus quickly replaced gas lasers in flow cytometers (Doornbos et al. 1994; Kapoor et al. 2008). The number of lasers installed in flow cytometers has increased rapidly, from two lasers in the mid-1990s to ten currently (Kapoor et al. 2007). The increase in laser lines, especially the addition of violet and ultraviolet lasers, has expanded the available excitation wavelengths, improving the multifluorescence excitation capability and the flexibility of parameter selection in flow cytometers (Shapiro and Perlmutter 2001; Telford and Frolova 2004).

Detectors are responsible for converting fluorescence signals separated by wavelength into photocurrent pulses. The number of detectors directly determines the number of parameters that can be simultaneously detected, while linearity and sensitivity determine the measurement range and signal resolution of each parameter. Most current commercial instruments employ high-gain photomultiplier tubes (PMTs) as fluorescence detectors (Buscher 2019). To further broaden the detection wavelength range and improve sensitivity, avalanche photodiodes (APDs) were introduced into flow cytometers in the first decade of the twenty-first century. APDs can work well in the near-infrared band of 800–1,000 nm and allow for the miniaturization of collection light paths and the integration of additional detectors (Lawrence et al. 2008).

The electronic system amplifies pulse signals from the detectors, measures pulses above the threshold, and it provides values for the pulse area (A), pulse height (H), and pulse width (W) (Snow 2004). The processing power of the electronic system affects the numerical resolution of the data and the analysis speed of the flow cytometer. Prior to the 2000s, flow cytometers used discrete analog circuits for signal processing and measurement (Xiong et al. 2015). Since the late 1990s, flow cytometers have instead used digital electronic systems, in which the amplified pulses of all detectors are digitized by analog-to-digital converters (ADCs) before processing, measurement, and computational full-matrix compensation, which simplifies the structure of the electronic system, reduces the footprint of the instrument, and facilitates the expansion of instrument architecture, thus enabling more complex functions (Zilmer et al. 1995).

Over its 50-year history, conventional flow cytometry has continued to evolve with the development of the optical and electronic technologies described above. Currently, the most powerful conventional flow cytometers are equipped with nine lasers and 60 fluorescence detectors, which are available from both BD Biosciences and Thermo Fisher. However, with the addition of fluorescent labels, the phenomenon of fluorescence spillover has become increasingly problematic (Mazza et al. 2018; McKinnon 2018). Fluorescence spillover significantly interferes with signal resolution and quantification accuracy, especially for weak signals (Nguyen et al. 2013; Njemini et al. 2014). This issue restricts the further addition of fluorochromes in one experiment (> 30) and complicates the design of multicolor flow cytometry experiments, requiring careful panel design and standardized workflows to ensure accuracy (Bagwell and Adams 1993; Futamura et al. 2015).

#### Spectral Flow Cytometry

Since the 1980s, researchers have been committed to analyzing the full fluorescence spectrum of cells to obtain additional biological information (Wade et al. 1979; Steen and Stokke 1986; Asbury et al. 1996; George et al. 2004). In 2004, spectral analysis was introduced into flow cytometry, and a continuous spectrum was generated from cell fluorescence using prism sets and acquired with a multichannel detector (Robinson et al. 2004; Nolan and Condello 2013). Using reference spectra obtained from standards as ‘fluorescent fingerprints’, the mixed fluorescence spectra from labels and intrinsic molecules can be mathematically deconvoluted to calculate the intensity of each signal, thus generating standard flow cytometry data (Robinson et al. 2004; Novo et al. 2013).

Compared with conventional flow cytometry, the greatest advantages of spectral flow cytometry are that the number of parameters and fluorescence channel selection are not constrained by the optical system and detectors, but spectral instruments retain full compatibility with the original experimental systems while giving unprecedented flexibility in experimental design and greatly reducing the complexity and manufacturing cost of instruments (Futamura et al. 2015). Spectral flow cytometry is capable of separating background signals from labels, which increases the specificity of the obtained fluorescence data (Robinson 2019). In addition, the raw data contain the full spectral information of a single cell, which can be mined for additional biological information in future studies. Currently, commercial spectral flow cytometers are available from Sony (Sony Biotechnology 2019), Cytek (Cytek Biosciences 2018) and Thermo Fisher (Thermo Fisher 2020). The latest Sony ID7000 model equipped with seven lasers can easily deconvolute over 40 fluorescent signals.

Since the success of spectral deconvolution depends entirely on the accuracy of spectra obtained from reference controls, any spectral changes to the fluorescence labels can result in deconvolution inaccuracies, which impair signal resolution and quantification or even result in deconvolution failure (Nolan and Condello 2013; Schmutz et al. 2016). Therefore, spectral flow cytometry is not suitable for fluorescent labels with unstable (e.g., tandem dyes) or variable (e.g., ratiometric probes) emission spectra. This limitation could be addressed by the development of novel standards or deconvolution algorithms.

#### Mass Cytometry

To fundamentally overcome the limitations of conventional flow cytometry and to adapt to the rapidly changing milieu in phenotype research, a novel omics-based cytometry technology was developed in 2008. Mass cytometry revolutionizes the original labeling and detection-based systems by using stable lanthanide isotopes with different atomic weights as labels, sample ionization by inductively coupled plasma (ICP), a time-of-flight (TOF) detector to measure the mass-to-charge ratio (m/z) of labeled ions and conversion of the discrete TOF signals into intensity data for each mass channel to yield quantitative flow cytometry data (Bandura et al. 2009; Ornatsky et al. 2010).

The TOF detector has sufficient mass resolution to distinguish isotopes with adjacent mass numbers, thus completely eliminating limitations on the densification of detection channels and enabling the measurement of over 100 parameters with little spillover (Spitzer and Nolan 2016). The single-particle sensitivity of the TOF detector is far better than that of conventional photodetectors, and the very low abundance of labeling elements in natural cells ensures no background noise (Bendall et al. 2012). These advantages position mass cytometry as an essential tool for in-depth cell phenotyping and signaling pathway analysis (Bendall et al. 2011, 2014; Bjornson et al. 2013; Becher et al. 2014).

Mass cytometry was commercialized by DVS (now a part of Fluidigm) in the late 2000s as the CyTOF family, which is now in its third generation, named Helios. Although there are presently more than 40 isotope labels available, the high cost of isotopic labels greatly restricts the commercial sources of reagents and the flexibility of panel design (Spitzer and Nolan 2016; Olsen et al. 2019). Moreover, the sample preparation procedures for mass cytometry are more tedious and sensitive to experimental variations than conventional flow cytometry (Yang and Herold 2017).

### Developments in Fluorochrome Synthesis to Expand Phenotype Granularity

Fluorochromes are the core elements of signal detection in flow cytometry. Over 50 years of flow cytometry development, five fluorochrome families have emerged: organic small molecules, natural macromolecules, inorganic quantum dots (qDots), tunable small molecules, and fluorescent polymers (Chattopadhyay et al. 2012). Small organic molecules, represented by fluorescein and cyanine dyes, are the earliest conjugatable fluorescent labels used in flow cytometry (The and Feltkamp 1970; Cunningham 2010). In the 1980s, natural fluorescent macromolecules from algae, named phycoerythrin (PE), allophycocyanin (APC), and peridinin-chlorophyll-protein complex (PerCP), were introduced to support analysis with 2–4 color channels (Oi et al. 1982; Axberg et al. 1991). To achieve analysis with > 5 colors, tandem derivatives of these early macromolecules were developed in 1995, which produce longer emission wavelengths through resonance energy transfer between a core fluorophore and coupling molecules, thus supplementing the selection of far red and near infrared fluorescent labels (Waggoner et al. 1993; Roederer et al. 1996). Tandem fluorochromes have wider emission spectra, lower brightness, and poorer stability than their core fluorophores and are usually used to detect abundant antigens (Chattopadhyay et al. 2012).

With the emergence of short-wavelength lasers and advances in chemical synthesis at the beginning of the twenty-first century, three new generations of fluorescent molecules were developed. Violet laser-excited qDots were first developed, increasing the number of available channels to 18 colors in a single experiment (Perfetto et al. 2004; Chattopadhyay et al. 2006). qDots are nanometer-scale semiconductor microcrystals with easily controlled emission wavelengths, high fluorescence brightness, excellent photostability, and good compatibility with existing labels. One major drawback is that coupling qDots with biological macromolecules is still challenging (Chattopadhyay et al. 2010). Tunable small molecules are a class of synthesized small fluorescent molecules with wavelength-tunable structures, including the well-known AlexaFluor and DyLight families. They have excellent stability and adequate fluorescent strength for flow cytometry and thus can replace some dimmer fluorochromes, such as FITC and APC (Chattopadhyay et al. 2012). Fluorescent polymers are a class of synthesized macromolecules containing linearly repeated fluorescent units (monomers) (Swager and Zhou 1995; Skotheim and Reynolds 2007). All fluorescent units in one molecule are coordinated during excitation and emission, conferring an an unprecedentedly high molar extinction coefficient superior to those of natural macromolecules and qDots (Chattopadhyay et al. 2012). Fluorescent polymers have good stability and high quenching resistance. They have been widely used in multicolor flow cytometry and have become the standard fluorescent label choice under violet and ultraviolet lasers. Current commercialized products include BD’s Brilliant Violet (BD Biosciences 2016a) and Brilliant Ultraviolet (BD Bioscience 2016b) and Thermo Fisher’s SuperBright series (Thermo Fisher 2018).

### Dimensionality-Reduction Algorithms Enable Visual Interpretation of High-Parameter Cell Phenomic Data

Analysis of conventional flow data requires scatter plots to simultaneously display two parameters (Herzenberg et al. 2006). For multicolor/spectral/mass flow data with dozens of parameters, it is necessary to draw tens to hundreds of plots to adequately convey the data. In addition, hierarchical gating analysis based on scatter plots may overlook the correlation information between parameters and meaningful unexpected cell phenotypes (Amir el et al. 2013).

To overcome the shortcomings of conventional analysis methods, multivariate analysis algorithms have become increasingly popular for flow cytometry data. One class of multivariate algorithms is clustering algorithms, represented by SPADE and FlowSOM (Qian et al. 2010; Qiu et al. 2011; Van Gassen et al. 2015). These algorithms concatenate the parameters of each cell and then cluster cells into nodes based on their overall similarity, resulting in a tree plot. One limitation of clustering algorithms is that cells with similar phenotypes are forced to merge into one node with only the average phenotype within the node displayed, thus reducing the granularity of the data (Amir el et al. 2013). An alternative to clustering algorithms is principal component analysis (PCA), which was introduced in 2011 for the analysis of mass spectrometry data, and it is a dimensionality reduction method that degenerates closely related variables and reduces information loss (Bendall et al. 2011). Nevertheless, PCA does not faithfully retain the characteristic nonlinear relationships in multiparametric datasets since it is based on linear transformation.

Since the late 2000s, dimensionality reduction algorithms based on stochastic neighbor embedding (SNE) have been introduced to analyze multiparametric data, the most popular of which are t-SNE (van der Maaten and Hinton 2008), vi-SNE (Amir el et al. 2013), and UMAP (Becht et al. 2018). Since these algorithms make use of nonlinear dimensionality reduction, they have good resolution for small-scale differences and can effectively distinguish cell populations with similar phenotypes. However, t-SNE and vi-SNE cannot accurately portray large-scale differences and are computationally time-consuming (van der Maaten and Hinton 2008; Lucchesi et al. 2020). An alternative to these algorithms is UMAP, which preserves larger-scale information and improves calculation speed while retaining the advantages of the t-SNE algorithm with higher efficiency of expressing phenotypic continuity (Becht et al. 2018; Pedersen and Olsen 2020).

## Design of a Multicolor Flow Cytometry Experiment for Cell Phenomics

Multicolor flow cytometry enables highly detailed and comprehensive analysis of cell phenomics, especially for situations with > 20 parameters. With the addition of parameters, the design of a multicolor flow cytometry experiment should comprehensively consider the instrument configuration (lasers, detectors, filters, and mirrors), reagents, spectral overlap, appropriate experimental controls, and quality control (QC) (Solly et al. 2019).

### Instrument Configurations

Flow cytometers that are equipped with optical splitting modules have the ability to detect several fluorochromes simultaneously. In such configurations, the light from fluorochromes is dissected by wavelength via several dichroic mirrors. To avoid loss of fluorescence during light transmission, it is extremely important to carefully examine the mirror’s transmission power (Perfetto et al. 2012). Dichroic mirrors are able to reflect fluorescence below the cutoff wavelength and to transmit fluorescence with a higher wavelength, or vice versa. In general, the cutoff wavelengths of dichroic mirrors are set somewhere between those of two fluorescence channels. For example, a 560 nm dichroic mirror is suitable for the detection of FITC (maximum emission at 520 nm) and PE (maximum emission at 590 nm). The sequential introduction of dichroic mirrors in optical settings allows for optimal separation and simultaneous detection of multiple fluorochromes.

The filters in front of the PMT that serve to collect light from dichroic mirrors are equally important. The choice of filter depends upon the emission wavelength of the fluorochrome measured. The passband of these filters should be wide enough to collect the maximum light arriving from the excited fluorochrome but never too wide to avoid transmitting of noncorresponding fluorescence. For example, for fluorescein (FITC) and PE excited by a blue laser (488 nm), the suggested filters for detection are 530/30 nm and 585/42 nm, respectively.

### Fluorescent Reagents and Staining

The selection of fluorescent reagents is critical for multicolor panel design. The brightness of the fluorochrome, the relative abundance of the antigen of interest, and the fluorescence compensation among the different channels are three main aspects that should be considered when choosing antibody conjugates.

For large multicolor panels, antigen density and relative fluorochrome brightness should be inversely matched. Specifically, lower density antigens (such as IL-4, IL-12, CXCR5, and CCR7) should be labeled with brighter fluorochromes (such as PE, PE-Cy5, PE-Cy7, and APC) for maximum resolution. For example, PE-conjugated monoclonal antibodies (mAbs) are suitable for detection of the expression of CD127 (the IL-7 receptor alpha-chain) on CD3^+^ T cells, while APC-conjugated mAbs are not (Lugli et al. 2009). Moreover, higher density antigens (CD3, CD4, CD8, CD20, and CD45) should be labeled with dimmer fluorochromes (eFluor 450, PerCP, and APC-Cy7) to generate a suitable signal intensity (Maecker et al. 2004).

The emission spectra of fluorochromes are wide, and their fluorescence in the selected channel can be inferred by the fluorescence from other fluorochromes. Another important consideration is therefore the spectral overlap of each fluorochrome and the amount of compensation needed. Compensation can subtract the unwanted fluorescence overspill in a specific channel but also affects the signal resolution of that channel. Whenever possible, the fluorochromes in an experimental panel should be distributed on multiple lasers to avoid overspill between adjacent channels and minimize compensation (Novo et al. 2013). To compensate correctly, samples that are stained with a single fluorochrome should be prepared. For tandem dyes, the conjugation ratio between the core fluorophore and conjugated molecules can be extremely variable and affect compensation. For this reason, the compensation matrix should always be checked when using a different batch of fluorophore-conjugated antibodies.

The antibody concentration is an important variable to consider in multicolor flow cytometry experiments. Insufficient antibody levels cannot fully occupy targets on the cell surface and may not adequately separate positive cells from negative ones, while excessive antibody levels promote nonspecific binding. To improve data resolution and produce robust results, antibodies used in multicolor flow staining should be titrated for their optimal concentration. In a typical titration, samples with the same cell type and number are stained with serially diluted antibody concentrations. In the results, the concentration that shows the best separation between the positive and negative populations should be regarded as optimal (Hurley 2001). To avoid result inconsistency caused by interlot diversity of antibodies, it is necessary to reperform titration after switching to a new lot.

Staining controls are increasingly important for complex multicolor flow cytometry experiments. To set up a new experiment, an unstained or blank control is used to adjust detector voltages, while single-positive controls are required to set the compensation matrix. To precisely gate a positive population out of a negative one, isotype controls and fluorescence-minus-one (FMO) controls are required to ensure the specificity of the signal. Isotype controls are used to identify nonspecific signals, which should have the same isotype, label, and labeling level, and it should be used at the same concentration as the antigen-specific antibodies (Wang and Hoffman 2017). By staining all antibodies except the one of interest, FMO controls allow the identification of background signals produced by fluorescence spillover and are used as a gating control (Maecker and Trotter 2006).

### Quality Control and Multicenter Harmonization

Since multicolor flow cytometry is susceptible to variations in sample quality, hardware performance, and experimental operations, QC is critical at each step to validate the reliability and reproducibility of the results. Instrument QC is an important preanalysis step during flow cytometry analysis and has been thoroughly discussed elsewhere (Hurley 2001; Perfetto et al. 2012). Instrument QC should be performed on a regular basis to optimize and normalize the performance of flow cytometers. This procedure consists of three steps: (i) system optimization to maximize sensitivity and signal resolution, (ii) instrument calibration to determine the dynamic range and photon efficiency of specific PMT detectors, and (iii) performance validation to test whether the data quality meets reproducibility requirements (Hurley 2001; Maecker and Trotter 2006; Perfetto et al. 2012). For most commercial instruments, the instrument QC procedure can be performed automatedly, with calibration beads providing standard fluorescent signals with specific intensities.

To reduce uncertainty from manual operations, automated liquid handling was first introduced into sample preparation. This advance enabled precise and parallel sample processing with ‘no wash’ reagents (Kelliher et al. 2005). Next, centrifugation was integrated into automation workstations to clear unbound antibodies (Wang et al. 2018; Wilson et al. 2018). Recently, centrifuge-less wash technology has been developed to avoid unwanted mechanical stimulation and cell loss produced by centrifugation (BD Biosciences 2012). Appropriate QC for sample preparation must be established and validated along with standard operating procedures (SOPs). First, cell viability and integrity in the samples should be preexamined to minimize nonspecific binding or artifacts caused by dead cells or debris (Muccio et al. 2018). Second, the number of cells in each sample should be adjusted to maintain an identical ratio between the cell number and antibody dosage (Owens et al. 2000). Finally, to test the validity of the staining panel, a standard sample should be carefully chosen; for example, whole-blood control samples with normal frequencies of lymphocyte subsets, which are available from BD Bioscience, Beckman Coulter, R&D, etc., can be used to test multicolor immunophenotyping panels (Owens et al. 2000; Wang and Hoffman 2017).

To improve the reproducibility of multicolor flow cytometry in multicenter cooperative studies, a workflow termed “multicenter harmonization” has been described to normalize data from multiple institutes so that a unified analysis strategy can be adopted in geographically distant laboratories (Le Lann et al. 2020). In this workflow, all involved flow cytometers are calibrated periodically using the same standard beads as an identical reference (Glier et al. 2019; Cornel et al. 2020). After collection, datasets from each instrument are normalized to the references to minimize the impact of performance fluctuations (Jamin et al. 2016). Finally, the mean fluorescence intensities (MFIs) of each parameter are aligned among all datasets to correct measurement disparities in the fluorescence among instruments while preserving individual variation for further analysis (Jamin et al. 2016; Le Lann et al. 2020). The implementation of multicenter harmonization enables large-scale statistical analysis of phenomics data, revealing the potential of multicenter analysis regardless of coverage, duration, sample size, and instruments required.

## Application of Multicolor Flow Cytometry for Cell Phenomics

Multicolor flow cytometry has the unique potential to analyze multiple parameters simultaneously at the single-cell level. It has thus been widely adopted to classify blood disorders (Woo et al. 2014), evaluate immune function (Abdolalipour et al. 2020), phenotype tumor cells (Nederlof et al. 2021) and assess immunotherapies (Bommareddy et al. 2019; Bonilla et al. 2020).

During differentiation and maturation, leukocytes display characteristic patterns of surface antigens when differentiating into different linages (Brown and Wittwer 2000; Craig and Foon 2008; Spijkerman et al. 2019). Even after transformation into leukemia cells, they generally retain their lineage-specific antigen profile but also show altered expression of some proteins that are rarely present in normal leukocytes. Such phenotypes are called leukemia-associated immunophenotypes (LAIPs). By combining the detection of multiple LAIPs in one panel, multicolor flow cytometry enables quick and precise classification of leukemia. As early as 2001, Weir and Borowitz (2001) summarized the LAIPs of most acute leukemia subtypes and described their corresponding flow cytometry panels. Recently, Porwit and Bene (2019) discussed the multicolor panels used in the diagnosis of mixed phenotype acute leukemias (MPALs), which is a rare subtype of acute leukemias with a poor prognosis. Rawstron et al. (2018) provided a consensus-recommended panel consisting of 14 markers for chronic lymphocyte leukemia (CLL) diagnosis after surveying the flow cytometry results of over 150 European patients.

In tumor immunity, multicolor flow cytometry has been widely used for the evaluation of immune functions before therapy selection to reveal the constitution and function of the immune system. Moncunill et al. (2015) published a report on a 16-color panel for thoroughly evaluating the responses of human T cell and NK cell subsets, including CD8^+^ T, CD4^+^ T, Th1, Th2, naïve T, memory T, effector T and follicular helper T cells. Tumor cells can evade the immune response by losing the expression of tumor antigens and/or actively inhibiting the responses of effector cells via inhibitory ligands, such as CTLA-4 and PD-L1 (Davoli et al. 2017; Juneja et al. 2017). Belkina and Snyder-Cappione (2017) provided a description of another 16-color panel to analyze the expression of inhibitory/exhaustive molecules on the surface of immune cells. At the design stage of therapy, multicolor flow cytometry is able to provide tumor immunophenotype (Davidson-Moncada et al. 2018) and local immune microenvironment information (Bommareddy et al. 2019; Garber 2019; Ahn et al. 2020) for the selection of therapeutic targets. It is also used to track the temporal heterogeneity of tumor cells to optimize treatment during cancer progression. In addition, multicolor flow cytometry can be used to monitor minimal residual diseases (MRDs) in the body after tumor remission and provide important data to support tumor prognosis (Bjorklund et al. 2009; Theunissen et al. 2017; Modvig et al. 2021).

### Perspectives

In this review, we aimed to provide a foundational overview of the advances in multicolor flow cytometry and its current state in phenotyping human cells, especially the immune and tumor cells. Multicolor flow cytometry is currently one of the best suited technologies for the detailed screening of various immunological parameters in blood cells. The drive towards industrialization of multicolor flow cytometry for analyzing cell phenomics of diseased tissue and organoid models will bring exciting prospects for disease classification and personalized medicine. In the future, supported by close collaborations between basic and clinical researchers, as well as industry partners, multiparametric flow cytometry assays will offer additional possibilities for the dynamic description of cell phenotypes.

## Data Availability

Not applicable.
